# Five Hidden Species in a Widespread European Vertebrate: Disentangling the Alpine Newt Cryptic Species Complex Through Genomic Phylogeography

**DOI:** 10.1111/mec.70300

**Published:** 2026-03-09

**Authors:** Stephanie Koster, Anagnostis Theodoropoulos, Wouter Beukema, Johanna Ambu, Wiesław Babik, Daniele Canestrelli, Andrea Chiocchio, Dan Cogălniceanu, Milena Cvijanović, Manon C. de Visser, Christophe Dufresnes, James France, Alban Hyseni, Daniel Jablonski, Daria Kranželić, Simeon Lukanov, Iñigo Martínez‐Solano, Borislav Naumov, Maciej Pabijan, Daniele Salvi, Bruno Schmidt, Konstantinos Sotiropoulos, Florina Stănescu, David Stanković, Emina Šunje, Márton Szabolcs, Emiliya Vacheva, Judit Vörös, Adnan Zimić, Ben Wielstra

**Affiliations:** ^1^ Institute of Biology Leiden Leiden University Leiden the Netherlands; ^2^ Naturalis Biodiversity Center Leiden the Netherlands; ^3^ RAVON (Reptile, Amphibian and Fish Conservation Netherlands) Nijmegen the Netherlands; ^4^ Laboratory for Amphibian Systematics and Evolutionary Research, College of Biology & the Environment Nanjing Forestry University Nanjing China; ^5^ Institute of Environmental Sciences, Faculty of Biology Jagiellonian University Kraków Poland; ^6^ Department of Ecological and Biological Sciences University of Tuscia Viterbo Italy; ^7^ Research Center of the Natural Sciences Department Ovidius University of Constanţa Constanţa Romania; ^8^ Department of Evolutionary Biology, Institute for Biological Research “Siniša Stanković” National Institute of Republic of Serbia, University of Belgrade Belgrade Serbia; ^9^ Blijdorp Conservation & Science Center Royal Rotterdam Zoological & Botanical Gardens Rotterdam the Netherlands; ^10^ Institut de Systématique, Evolution, Biodiversité (ISYEB), Muséum National D'histoire Naturelle, CNRS, Sorbonne Université, EPHE‐PSL Université Des Antilles Paris France; ^11^ Department of Biology, Faculty of Mathematical and Natural Sciences University of “Hasan Prishtina” Prishtina Kosovo; ^12^ Department of Zoology Comenius University in Bratislava Bratislava Slovakia; ^13^ Association Hyla, Lipovac I No. 7 Zagreb Croatia; ^14^ Institute of Biodiversity and Ecosystem Research Bulgarian Academy of Sciences Sofia Bulgaria; ^15^ Museo Nacional de Ciencias Naturales Madrid Spain; ^16^ Department of Comparative Anatomy, Institute of Zoology and Biomedical Research, Faculty of Biology Jagiellonian University Kraków Poland; ^17^ Department of Health, Life & Environmental Sciences University of L'aquila L'Aquila‐Coppito Italy; ^18^ Public Institution for the Management of Protected Areas and Other Protected Parts of Nature at the Zagreb County Level “Zeleni Prsten” Samobor Croatia; ^19^ Molecular Ecology and Conservation Genetics Lab, Department of Biological Applications & Technology University of Ioannina Ioannina Greece; ^20^ Natural Sciences Department, CEDMOG Ovidius University of Constanţa Romania; ^21^ National Institute of Biology Ljubljana Slovenia; ^22^ Evolutionary Ecology Group, Faculty of Science University of Sarajevo Sarajevo Bosnia and Herzegovina; ^23^ Herpetological Association in B&H, ATRA (BHHU ‐ATRA) Sarajevo Bosnia and Herzegovina; ^24^ Laboratory of Functional Morphology University of Antwerpen Wilrijk Belgium; ^25^ HUN‐REN Centre for Ecological Research Institute of Aquatic Ecology, Conservation Ecology Research Group Debrecen Hungary; ^26^ HUN‐REN Balaton Limnological Research Institute Hungary; ^27^ Herpetological Association in Bosnia and Herzegovina – Atra Sarajevo Bosnia and Herzegovina; ^28^ National Museum of Bosnia and Herzegovina Sarajevo Bosnia and Herzegovina

**Keywords:** ghost lineage, hybridisation, *Mesotriton alpestris*, speciation, target sequence capture, taxonomy

## Abstract

Through genomic phylogeography, previously unrecognised biodiversity can be revealed. The alpine newt serves as a case in point: this taxon carries highly distinct mtDNA clades and has a severely fragmented range. We obtain genome‐wide data with target enrichment by sequence capture to delineate cryptic species and disentangle their phylogenetic relationships. Furthermore, we explore potential niche divergence and glaciation‐driven distribution dynamics. On the basis of the uncovered genetic structure, we distinguish five main groups that we propose should be treated as distinct species. Limited interspecific genetic admixture often occurs away from current contact zones between these species, in line with a scenario of current range reduction, compared to the Last Glacial Maximum. A decline in suitable habitat also explains the fragmented nature of current species ranges. We uncover pronounced mito‐nuclear discordance. We show that an ancient mtDNA lineage endemic to the Vlasina Plateau on the border between Serbia and Bulgaria, previously interpreted to be a ‘ghost lineage’, in fact represents a distinct species. However, it is nested considerably deeper inside the alpine newt species complex than mtDNA suggests. Our study illustrates how genomic phylogeography allows intricate evolutionary histories to be untangled.

## 1 Introduction

Cryptic species are – or until recently have been – treated as a single species, due to their morphological similarity, but are inferred to represent evolutionary independent lineages based on genetic data (Beheregaray and Caccone 2007; Bickford et al. 2007; Espíndola et al. 2016; Fišer et al. 2018; Hending 2025; Pfenninger and Schwenk 2007; Struck et al. 2018). Morphology‐based taxonomy is biased towards human visual perception, which is rather limited in capturing the entire range of biological complexity. The corollary is that biodiversity has long been underestimated. With the advent of genetic data, it has become apparent that cryptic biodiversity is rampant and genetic data are playing an increasingly important role in identifying and delineating species (Beheregaray and Caccone 2007; Bickford et al. 2007; Espíndola et al. 2016; Fišer et al. 2018; Hending 2025; Pfenninger and Schwenk 2007; Struck et al. 2018).

The majority of cryptic species have been hypothesised based on single DNA markers, particularly mtDNA (Fišer et al. 2018). While mtDNA is quite efficient in identifying potential cryptic species (Avise 2000; Hebert et al. 2003), it also has well‐known limitations (Ballard and Whitlock 2004; Balloux 2010; DeSalle 2006; Moritz and Cicero 2004). Geographical populations bearing distinct mtDNA lineages may show unimpeded nuclear gene flow (Benham and Cheviron 2019; Dufresnes et al. 2020; Hinojosa et al. 2019; Hogner et al. 2012; Irwin 2002; Mao et al. 2013; Pereira and Wake 2009; Wielstra et al. 2021; Zhang et al. 2019). Even if mtDNA lineages do reflect distinct species, introgression of mtDNA regularly causes the distribution of these lineages to deviate from the species boundary as defined by nuclear DNA (Bonnet et al. 2017; Chan and Levin 2005; Currat et al. 2008; Petit and Excoffier 2009; Toews and Brelsford 2012; Wielstra 2019).

Genome‐wide molecular data are crucial for testing the evolutionary independence of putative cryptic species (Avise 2000; Dufresnes et al. 2023; Edwards 2009; Vences et al. 2024). Advances in DNA sequencing techniques have made it possible to genotype large numbers of individuals, for many markers scattered across the genome, at a reasonable cost (Ekblom and Galindo 2011; Garrick et al. 2015; McCormack et al. 2013; Twyford and Ennos 2012). Nowadays, genome‐wide molecular data are regularly employed to delineate cryptic species (Dufresnes and Jablonski 2022; Hinojosa et al. 2019; Janzen et al. 2017; Kalaentzis et al. 2023; Weiss et al. 2018; Wielstra and Arntzen 2016).

Target enrichment by sequence capture is a particularly suitable approach for consistently sampling the same set of genome‐wide markers (Andermann et al. 2020; Gnirke et al. 2009; Grover et al. 2012; Jones and Good 2016). Sheared, indexed and size‐selected genomic DNA is hybridised to RNA baits that are complementary to the DNA of interest. The baits bind to the target DNA and are subsequently captured with magnetic beads, while the rest of the DNA is washed away, allowing the target DNA to be sequenced. A particular advantage of target enrichment by sequence capture is that a perfect match between bait and target sequence is not required, enabling a single set of baits to capture DNA from populations or species that are genetically diverged (Bi et al. 2012; Bragg et al. 2016; de Visser, France, McCartney‐Melstad, et al. 2025) – as would be expected in a cryptic species complex.

The integration of ecological analyses within phylogeography provides additional guidance for disentangling cryptic species complexes – although care should be taken not to overinterpret the results (Barve et al. 2011; Journé et al. 2020; Soberon and Peterson 2005). Through species distribution modelling, past range dynamics such as range fragmentation or fusion can be inferred (Kozak et al. 2008; Svenning et al. 2011). This can be used to explore the drivers of mito‐nuclear discordance, for example by the engulfment of a glacial relict population during postglacial range expansion (Wielstra et al. 2021), mitochondrial haplotype surfing following demographic changes involving admixed populations (Dufresnes et al. 2025, 2019), or species replacement with hybridisation upon secondary contact, that is hybrid zone movement (Wielstra and Arntzen 2012). Furthermore, quantification of bioclimatic niche overlap can be used to test potential ecological differences among evolutionary lineages delineated through genomic phylogeography (Espíndola et al. 2016; Hending 2025). For example, it could be tested if genetically defined populations would respond differently to environmental change (Čengić et al. 2024), or if potential cryptic species show differences in the ecological conditions they inhabit that are comparable to those among recognised species (Wielstra et al. 2012).

**FIGURE 1:**
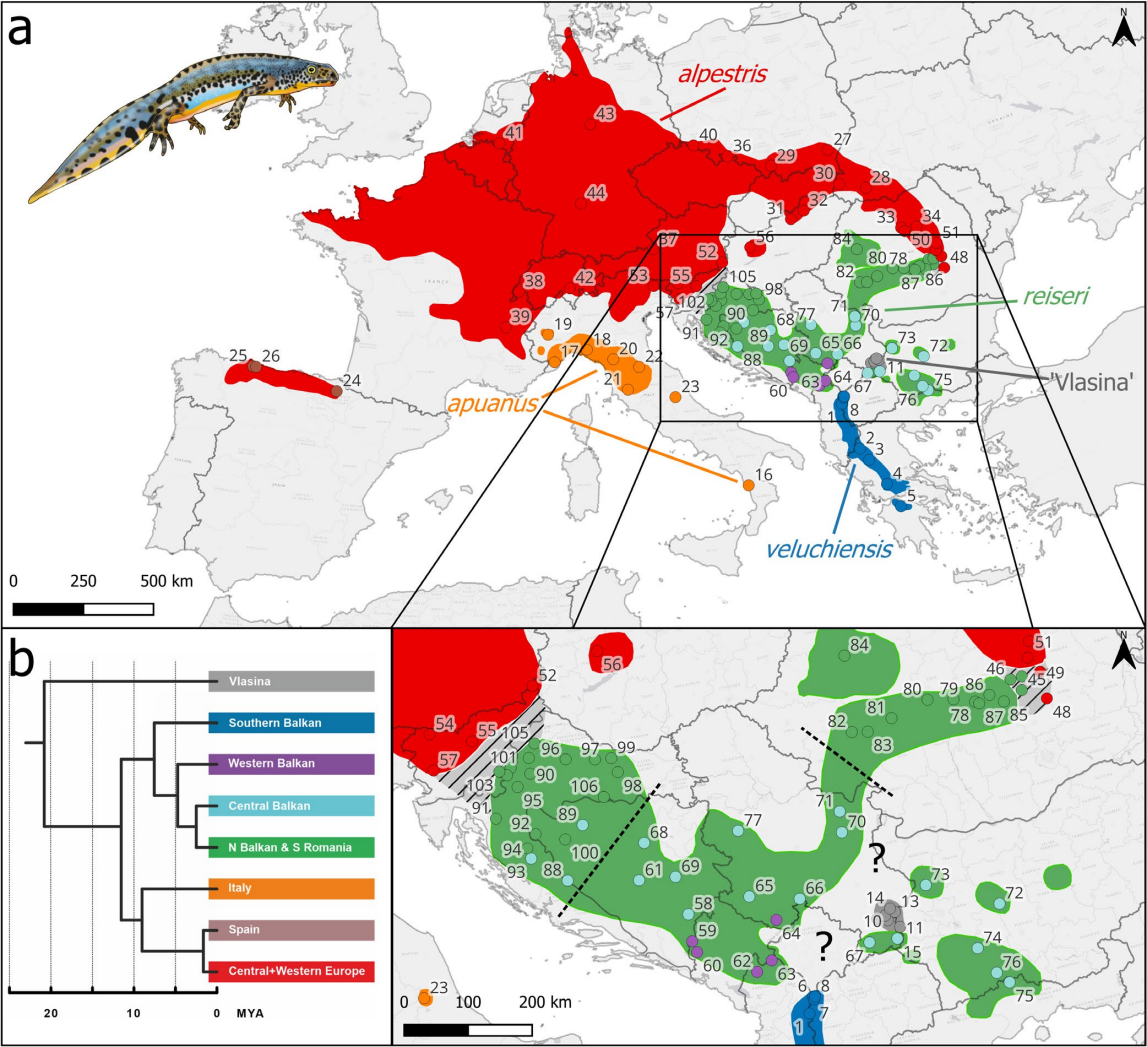
Distribution and mtDNA phylogeny of the alpine newt (*Mesotriton*) cryptic species complex. In (a) the background colour reflects the ranges of the five nuclear DNA groups comprising the alpine newt that we propose to recognise as species (see Discussion, Section 4.5). Dotted lines mark the approximate boundaries between nuclear DNA subgroups within *reiseri*. Numbered dots show the sampled localities (Table S1) and are colour‐coded according to mtDNA clade. In (b) a simplified, dated mtDNA phylogeny is depicted (based on Recuero et al. 2014). The ‘N Balkan & S Romania’ mtDNA clade encompasses samples from the Northern Balkan and Southern Romania nuclear DNA subgroups. The ‘Western Balkan’ mtDNA clade is not clearly reflected by a nuclear DNA subgroup.

The advances in phylogeography outlined above facilitate the testing of cryptic species hypotheses raised in mtDNA‐based studies. The alpine newt ( *Mesotriton alpestris* ), a cold‐adapted amphibian whose currently fragmented range stretches across much of Europe (Figure 1a), exemplifies the need for integrating genome‐wide and ecological data in cryptic species delineation. On the basis of the presence of multiple, vastly distinct mtDNA clades (Figure 1b), this taxon is strongly suspected to represent a cryptic species complex (Recuero et al. 2014; Robbemont et al. 2023; Sotiropoulos et al. 2007). Yet, while extremely limited nuclear DNA data suggest that gene flow is restricted between some of these mtDNA clades, it appears to be unimpeded between other mtDNA clades (Recuero et al. 2014). Therefore, taxonomic authorities have been reluctant to partition the alpine newt into multiple species (Frost 2025; Speybroeck et al. 2020). We apply target enrichment by sequence capture and incorporate ecological analyses to unravel the intricate evolutionary history of the cryptic alpine newt species complex. Specifically, we determine (1) genetic structure, (2) genetic admixture, (3) phylogenetic relationships, (4) molecular dating, (5) historical introgression and (6) ecological divergence. Our study demonstrates how advances in phylogeography can help uncover hidden biodiversity – crucial information in the effort to slow the Holocene extinction.

## 2 Materials and Methods

### 2.1 Samples, DNA Extraction and mtDNA

We included 106 *Mesotriton* samples, from the same number of localities, distributed throughout the entire range (Figure 1a, Table S1). We took 78 DNA extractions from a previous study (Robbemont et al. 2023), and DNA from an additional 28 samples was extracted with the Promega Wizard Genomic DNA Purification Kit (Table S1). The outgroup included three individuals for each of the four species *Calotriton asper* , *Triturus carnifex* , *T. macedonicus, T* *. marmoratus* (de Visser, France, Paulouskaya, et al. 2025; Kazilas et al. 2024; Wielstra et al. 2019). Individuals not previously mtDNA barcoded were assigned to mtDNA clade based on a 651‐bp stretch of the ND4 gene or, if that longer fragment failed, a 241‐bp ’internal’ ND4 fragment, as described in Robbemont et al. (2023). For three individuals that could not be genotyped by either method, the mtDNA clade to which they belong was inferred based on geography (Figure 1a, Table S1).

### 2.2 Target Sequence Capture and Bioinformatics

We used the NewtCap target sequence capture protocol (de Visser, France, McCartney‐Melstad, et al. 2025) to obtain genomic data. In brief, this encompassed preparation of indexed libraries with the NEBNext Ultra II FS DNA Library Prep Kit for Illumina (New England Biolabs, Ipswich, MA, USA). Library concentration and quality were determined with a Fragment Analyser (Agilent, Santa Clara, CA, USA). We then performed target enrichment using a custom probe set of 7139 targets based on *Triturus* transcriptome data (MyBaits v4.0 kit, Arbor Biosciences Ref# 170210‐32; Wielstra et al. 2019). Concentration and size distribution of the enriched libraries were analysed on the Agilent 4150 TapeStation system (Agilent, Santa Clara, CA, USA). Enriched libraries were pooled and sent to BaseClear B.V. (Leiden, the Netherlands) for 150 bp paired‐end sequencing on the NovaSeq 6000 platform (Illumina Inc., San Diego, CA, USA), with an aim to sequence 1 Gbp per sample.

A bioinformatics pipeline, described in detail in the NewtCap target sequence capture protocol (de Visser, France, McCartney‐Melstad, et al. 2025), was used to check the quality of the data and to clean, map and combine the reads. Trimmed reads were mapped to a reference set of 7139 *T. dobrogicus* sequences (Wielstra et al. 2019). A custom R script was used to determine sequence coverage for each sample (France et al. 2025). For the variants in the multi‐sample VCF resulting from the pipeline, Hardy Weinberg‐related statistics were calculated with BCFtools v1.15.1 (Danecek et al. 2021), and any variant sites with heterozygote excess (*p* < 0.05) were filtered out to reduce the effect of paralogues. Insertions/deletions and variant sites with a genotype quality below 20 or with missing data in more than 25% of samples were also filtered out, and a minor allele frequency filter of 0.05 was applied, using BCFtools and VCFtools v0.1.16 (Danecek et al. 2011). This filtered set was used for the Principal Component, ADMIXTURE, IQ‐TREE, wASTRAL and Dsuite analyses, while for the SNAPPER and TreeMix analyses, all sites with missing data were removed.

### 2.3 Genetic Structure: Principal Component Analysis and ADMIXTURE

We performed Principal Component Analysis in RStudio 2024.04.2 (RStudio Team 2024) in R 4.4.1 (R Core Team 2025), using the SNPRelate and gdsfmt (Zheng et al. 2012) R packages to visualise genetic structure within the alpine newt. We created a subset of SNPs, with one SNP per target randomly selected through a custom Perl script and then converted to BED format in PLINK v1.07 (Purcell et al. 2007), resulting in 3182 SNPs. We further explored genetic structure and admixture within the alpine newt using ADMIXTURE v1.3.0 (Alexander et al. 2009). We removed non‐variant and multiallelic sites using VCFtools, then used PLINK to remove loci with high linkage using a 1 Mb window, a 1 SNP step, and an r2 threshold of 0.8. VCFtools was used to remove these sites from the file, resulting in 29,268 SNPs. We ran ADMIXTURE for *K* = 1–20 with five replicates per *K*‐value, using cross‐validation to determine the optimal number of ancestral populations. We then ran ADMIXTURE for this optimal *K*‐value with 25 iterations and summarised the results in R.

### 2.4 Phylogenomics: Concatenated Analysis and Molecular Dating

Because the inclusion of genetically admixed individuals hampers phylogenetic inference (Ambu et al. 2023; Gippner et al. 2024), we excluded 49 individuals highlighted as such in the ADMIXTURE analysis (see Results, Section 3.3; Table S1) from our phylogenetic analyses; we took a conservative approach here and removed any individual that was not allocated to a particular ancestral population with a score ≥ 0.99. A concatenated maximum likelihood phylogenetic tree was inferred in IQ‐TREE v3.0.1 (Wong et al. 2025). To properly root the tree for divergence time estimations (see below), we included a single *C. asper* individual, the most distantly related species in the outgroup. To run a partitioned analysis (Chernomor et al. 2016), we split the VCF file into separate files for each target using SnpSift v4.3 (Cingolani et al. 2012), then converted the separate VCF files into PHYLIP format using the vcf2phylip.py Python script (Ortiz 2019). We then ran IQ‐TREE, first to create the partition file from the separated targets, resulting in 6935 partitions, and then inferred the phylogeny. We used the GTR + G model for all partitions and 1000 ultrafast bootstrap iterations (Hoang et al. 2018). The phylogeny was inferred based on 83,259 SNPs.

We conducted dated inference in treePL v2.6.3 (Smith and O'Meara 2012) and in MEGA 11.0.13 (Tamura et al. 2021) with the RelTime‐ML method (Mello 2018; Tamura et al. 2012). We employed two fixed calibration points: the split between *T. carnifex* and *T. macedonicus* , dated to 5.33 Ma, and the basal split between *T. marmoratus* and *T. carnifex* plus *T. macedonicus* , dated to 24 Ma (Steinfartz et al. 2007; Wielstra and Arntzen 2011). For treePL, a priming analysis was performed to obtain the optimal parameters for the final analysis (Maurin 2020). The optimisation parameters used were as follows: opt = 2, moredetail, optad = 3, moredetailad and optcvad = 5. The optimal smoothing value was 0.000000001. To add 95% confidence intervals to the phylogeny, we ran 1000 ultrafast bootstrap replicates in IQ‐TREE. The treePL analysis was repeated for each bootstrap replicate, and the trees were summarised in TreeAnnotator v2.4.7 (Bouckaert et al. 2019). For RelTime‐ML, we converted the VCF file used for IQ‐TREE to FASTA format using the vcf2phylip.py Python script (Ortiz 2019). *Calotriton asper* was set as the outgroup in the rooted IQ‐tree phylogeny and default settings were used for the analysis.

### 2.5 Phylogenomics: Species Tree Analyses

In our species tree analyses, we again excluded the 49 individuals highlighted as showing signs of recent genetic admixture in the ADMIXTURE analysis (see Results, Section 3.3; Table S1). A summary multi‐species coalescent‐based estimation of the phylogeny was produced with wASTRAL v1.23.3.7 (C. Zhang and Mirarab 2022). We split our VCF file into separate files for each target using SnpSift v4.3. The separate VCF files were converted to PHYLIP format as above. We then used IQ‐TREE to infer gene trees for the individual targets, employing the GTR + G model. The resulting 3027 gene trees were combined into a single file and used as input for wASTRAL. The allocation of *Mesotriton* individuals to eight nuclear DNA (sub)groups was guided by PCA, ADMIXTURE and IQ‐TREE (see Results Section 3.4; Table S1). We also used ASTRAL‐III v5.7.8 (C. Zhang et al. 2018) to calculate the normalised quartet score, based on the wASTRAL tree and the original gene trees.

A Bayesian species tree inference using a diffusion model was conducted in SNAPPER v1.1.3 (Stoltz et al. 2021). A VCF file with one randomly selected SNP for 2217 targets was generated with a custom Perl script. This file was then converted into binary NEXUS format using the Python script vcf2phylip.py (https://github.com/edgardomortiz/vcf2phylip), resulting in a file with 2180 SNPs (a slightly lower number because no missing data is allowed in this analysis). This binary NEXUS file was subsequently converted to XML format in BEAUti v2.7.5 in BEAST v2.7.4 (Bouckaert et al. 2019), with default parameters and a chain length of 10 million generations for three replicates. As before, individuals were allocated to eight *Mesotriton* nuclear DNA (sub)groups (see Results, Section 3.4; Table S1). We ran SNAPPER within BEAST and combined output files with LogCombiner v2.7.5 (Bouckaert et al. 2019), removing a 10% burn‐in from each replicate. The remaining phylogenies were then combined with TreeAnnotator v2.7.5 (Bouckaert et al. 2019). Convergence of the runs, as well as the effective sample sizes of the parameters, were checked in Tracer v1.7.2 (Rambaut et al. 2018).

### 2.6 Introgression Analyses With Dsuite and TreeMix

To explore historical introgression, we again excluded the 49 individuals highlighted as showing signs of recent genetic admixture in the ADMIXTURE analysis (see Results, Section 3.3; Table S1). We then selected one SNP per marker as above, and confirmed there were no sites with high linkage using PLINK v1.07 (Purcell et al. 2007) with settings indep‐pairwise 50 10 0.1. This VCF file, with outgroups and containing 6624 SNPs, was analysed in Dsuite v0.5‐r48 (Malinsky et al. 2021). The Dtrios function was used to calculate the *f4* admixture ratio statistic for all possible trios of the eight *Mesotriton* nuclear DNA (sub)groups (see Results, Section 3.4; Table S1), with the outgroup. Next, we used the Fbranch function to determine *f*‐branch statistics for all positive *f4* admixture ratios, based on the topology inferred by our phylogenomic analyses (see Results, Section 3.5). This approach allows genetic admixture signals to be assigned to specific (potentially internal) branches of the species tree (Malinsky et al. 2021). The results were visualised as a heatmap using the dtools.py Python script included with Dsuite. For TreeMix v1.13 (Pickrell and Pritchard 2012), we used the file without outgroups, leaving 2180 SNPs (a slightly lower number because no missing data is allowed in this analysis). With Stacks v2.64 (Catchen et al. 2013) each sample was assigned to the correct taxon and the file was converted to TreeMix format. We ran TreeMix for 0 to 10 migration edges, with 10 reticulations each, and used OptM v0.1.8 (Fitak 2021) to determine the optimal number of migration edges, based on the linear modelling estimation.

### 2.7 Species Distribution Modelling and Niche Overlap

We partitioned > 17 k native *Mesotriton* occurrences (Table S2) into the five primary nuclear DNA groups that we identified based on genome‐wide molecular data (see Results, Section 3.4). Localities that could not confidently be ascribed to only one of these five nuclear DNA groups, i.e., could be affected by genetic admixture with another nuclear DNA subgroup, were excluded (Table S2). We did not consider smaller subgroups that are phylogenomically nested within these five primary nuclear DNA groups, because these are relatively closely related and genetic admixture between them is geographically extensive where they meet in parapatry (see Results, Section 3.4).

We downloaded the full set of 19 WorldClim bioclimatic variables for current and Last Glacial Maximum conditions at approximately 1 × 1 km resolution (Brown et al. 2018; Fick and Hijmans 2017), which we restricted to a rectangular study area encompassing Europe south of 62° N latitude. Pairwise Pearson's *r* scores were calculated to assess the degree of multicollinearity between variable pairs using the ENMTools package in R (Warren and Dinnage 2025). Decisions on which variable to retain when a pair of variables displayed a Pearson's *r* higher than 0.7 or lower than −0.7 were made based on expert knowledge of *Mesotriton* ecology. The final subset comprised eight variables, summarising temperature‐ and precipitation extremes and temperature averages considered relevant to ectotherms, covering both the reproduction and aestivation seasons; Isothermality (bio03; (bio2/bio7)×100), Temperature Seasonality (in °C, standard deviation ×100; bio04), Max. Temperature of Warmest Month (in °C; bio05), Min. Temperature of Coldest Month (in °C; bio06), Mean Temperature of Wettest Quarter (in °C; bio08), Precipitation Seasonality (Coefficient of Variation, in mm; bio15), Precipitation of Warmest Quarter (in mm; bio18) and Precipitation of Coldest Quarter (in mm; bio19).

The machine‐learning species distribution modelling algorithm Maxent (Phillips et al. 2006) was used to generate suitability predictions for current conditions and the Last Glacial Maximum via the R package ENMeval (Kass et al. 2021). Maxent is one of the most widely used species distribution modelling algorithms, often outperforms other species distribution modelling methods, and generally achieves good results when using small datasets (e.g., Wisz et al. 2008). Applying Maxent in ENMeval permits splitting occurrence data into ‘spatial blocks’, which, among other benefits, decreases the chance of high spatial autocorrelation between training and testing sites, and thereby counters model overfitting (Valavi et al. 2019). ENMeval also includes model selection procedures (Morales et al. 2017). Occurrence‐ and background data were first divided into spatial blocks to decrease the chance for high spatial autocorrelation between training and testing sites. Then, a set of initial models was created for these subsets, each with different combinations of parameter settings, which together represent all combinations of regularisation multiplier (1–5) and feature class settings (‘linear’ (L), ‘linear & quadratic’ (LC), hinge (H), and linear, quadratic & hinge (LQH)). The optimal model was defined as the model with the lowest mean percentage of test sites falling outside the predicted range (i.e., lowest mean omission error), and the highest mean evaluation (AUC) value (Low et al. 2021; Velasco and González‐Salazar 2019). To check whether this model performed significantly better than models based on randomly selected data, a series of 100 null models was created (Bohl et al. 2019; Raes and ter Steege 2007). Plots of occurrence data divided into spatial blocks, metadata, species‐environment relationship plots, and the spatial predictions (suitability maps) for current conditions and the Last Glacial Maximum were exported as separate files.

Bioclimatic niche overlap between nuclear DNA groups was subsequently measured using ordination, as species distribution modelling‐based niche overlap approaches do not allow for the degree of model parameterisation described above. A Principal Component Analysis calibrated on the entire study area (PCA‐env; Broennimann et al. 2012) was used as basis for niche overlap measurements. The first two principal components were subsequently used to generate a 2D gridded representation of environmental space, in which climatic niches were plotted and overlap was calculated using Schoener's D and Hellinger's I statistics (Broennimann et al. 2012). These indices range from 0 (no overlap) to 1 (complete overlap; niche equality). Similarity (or background) tests were performed to compare the overlap between two nuclear DNA groups to the overlap expected by chance if one or both were effectively choosing habitat at random from within their broad geographic range. The purpose of this test is to correct for the availability of habitat, and ask whether the observed similarity between nuclear DNA groups is significantly more (or less) than expected, given the available set of environments in the regions in which they occur.

## 3 Results

### 3.1 Sequencing Success

The 129 Gb of raw sequence capture data obtained contains on average 14.7 (s.d. 10,3) million read pairs per sample. Of the filtered reads, on average 26.8% (s.d. 16.8%) map to our reference. The mean percentage of duplicate reads is 56.7% (s.d. 16.1%). The average number of targets that has at least one read is 7107 (s.d. 26.5), and the median peak 100 bp coverage of the samples is 45.7 (s.d. 27) on average, ranging from 11 to 135.

### 3.2 Genetic Structure

**FIGURE 2:**
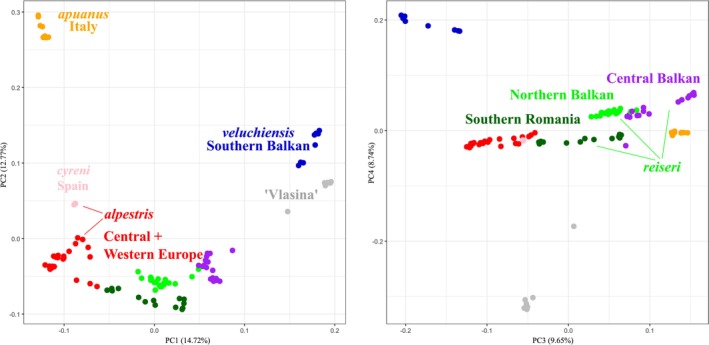
Principal Component Analysis of the alpine newt (*Mesotriton*) cryptic species complex. The first four principal components are shown. Colours correspond to the eight nuclear DNA (sub)groups identified in the ADMIXTURE analysis (Figure 3).

Our analyses reveal considerable genetic structure in the alpine newt. For the Principal Component Analysis we present results for the first four principal components (Figure 2). Geographical groups that stand out concern localities from the Southern Balkan Peninsula, the Italian Peninsula, the Vlasina Plateau (in eastern Serbia and the extreme west of Bulgaria), Spain, Central+Western Europe and the Balkan‐Carpathian region. The intermediate positions of a few samples can be explained by genetic admixture (see Results, Section 3.3).

**FIGURE 3:**
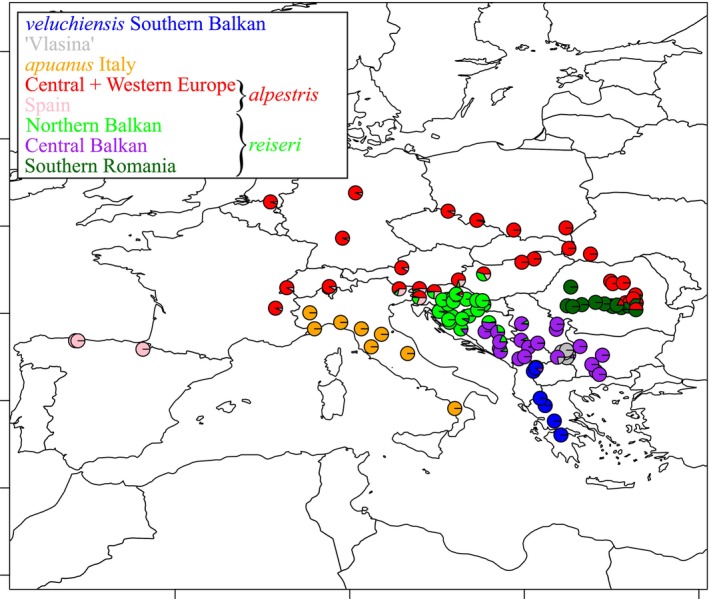
ADMIXTURE analysis of the alpine newt (*Mesotriton*) cryptic species complex. The number of ancestral gene pools (K) displayed is 8, the most likely number based on cross‐validation (Figure S1). Braces indicate the nuclear DNA subgroups that together correspond to *alpestris* (top) and *reiseri* (bottom). See Table S1 for details.

For ADMIXTURE we present the results under *K* = 8 ancestral gene pools (Figure 3; Table S1), the most likely number based on cross‐validation (Figure S1). Again, localities from the Southern Balkan Peninsula, the Italian Peninsula, the Vlasina Plateau, Spain and Central+Western Europe stand out as distinct genetic clusters. Furthermore, for the Balkan‐Carpathian region, localities from the Northern Balkan Peninsula, the Central Balkan Peninsula and Southern Romania comprise distinct genetic clusters.

**FIGURE 4:**
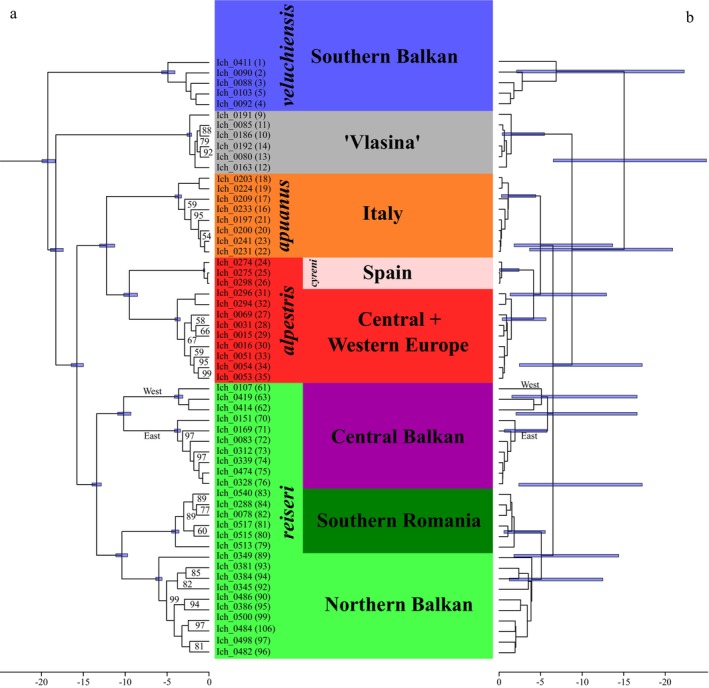
Dated concatenated maximum likelihood phylogenetic tree of the alpine newt (*Mesotriton*) cryptic species complex, inferred with (a) treePL and (b) RelTime. The treePL 95% confidence intervals are calculated using 1000 IQ‐TREE ultrafast bootstrap phylogenies. The RelTime 95% confidence intervals are estimated by the program. Confidence intervals are only shown for major bifurcations; those within nuclear DNA (sub)groups are not shown. All nodes have 100% bootstrap support unless otherwise indicated (only in panel a). The scale bar shows branch ages in five‐million‐year intervals.

The concatenated maximum likelihood analysis with IQ‐TREE recovers the same genetic clusters as in the ADMIXTURE analysis as monophyletic groups (Figure 4). We see clades comprising localities from the Southern Balkan Peninsula, the Italian Peninsula, and the Vlasina Plateau. Clades comprising localities from Spain and Western+Central Europe cluster together and so do clades comprising localities from the Northern Balkan Peninsula, the Central Balkan Peninsula, and Southern Romania. Furthermore, the Central Balkan Peninsula clade comprises a Western and an Eastern component that we only recover in ADMIXTURE under *K* = 9, which exhibits slightly higher cross‐validation errors compared to *K* = 8 (Figures S1 and S2).

### 3.3 Genetic Admixture

Our ADMIXTURE analysis under *K* = 8 exposes local genetic admixture between nuclear DNA groups, at the scale of tens of kilometres (Figure 3; Table S1). In the northernmost part of the range of *veluchiensis*, we observe genetic admixture with *reiseri*. In the southeast of Serbia, genetic admixture between ‘Vlasina’ and *reiseri* occurs. Genetic admixture between *alpestris* and *reiseri* is found at their western (Slovenia, northern Croatia and western Hungary) and eastern (southeastern Carpathians in Romania) contacts. There is no genetic admixture with *apuanus*.

Genetic admixture between nuclear DNA subgroups is relatively extensive, that is at the scale of hundreds of kilometres (Figure 3b; Table S1). Substantial genetic admixture between the Central+Western Europe and Spain nuclear DNA subgroups of *alpestris* is observed in Slovenia and adjacent Hungary and Italy. Less extensive genetic admixture is seen across most of the range, in particular across Western Europe. Two of the three nuclear DNA subgroups comprising *reiseri*, Central Balkan and Northern Balkan show considerable genetic admixture where they meet each other; the allopatrically distributed Southern Romania nuclear DNA subgroup shows no such genetic admixture with the other *reiseri* nuclear DNA subgroups.

### 3.4 Nuclear DNA (Sub) Groups

Taken the above analyses together (see Results, Sections 3.2 and 3.3), we recognise five primary nuclear DNA groups that we refer to as (1) Southern Balkan *veluchiensis*, (2) ‘Vlasina’ from the Vlasina Plateau, (3) *apuanus* from Italy, (4) *alpestris* from Central+Western Europe & Spain, and (5) *reiseri* from the Balkan‐Carpathian region (Figure 1a). These are the clades that are genetically most distinct in the concatenated maximum likelihood analysis and that express geographically limited genetic admixture. Two of the five primary nuclear DNA groups can be further subdivided into nuclear DNA subgroups: *alpestris* in (4a) Central+Western Europe and (4b) Spain; and *reiseri* in (5a) Central Balkan, (5b) Northern Balkan, and (5c) Southern Romania. These comprise relatively closely related clades in the concatenated maximum likelihood analysis and express extensive genetic admixture where they meet in parapatry. We use these nuclear DNA (sub) groups in downstream analyses.

### 3.5 Phylogenomics

**FIGURE 5:**
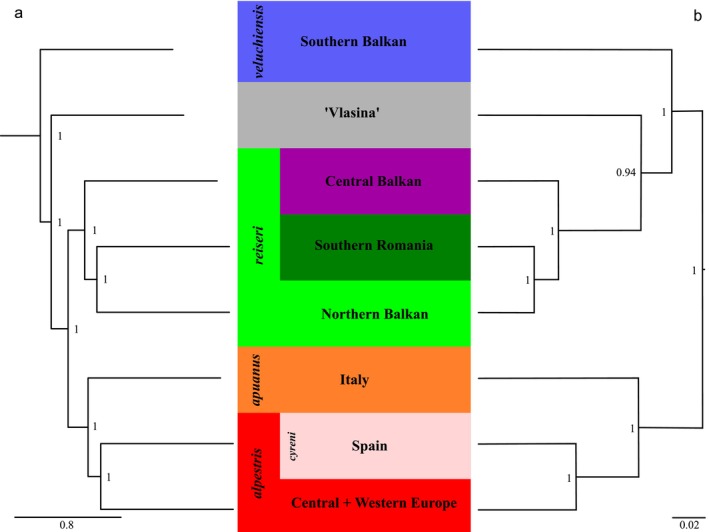
Species trees of the alpine newt (*Mesotriton*) cryptic species complex. (a) Summary multi‐species coalescent‐based estimation with wASTRAL. Branch lengths represent coalescent units and support values are local posterior probabilities. The outgroup is not shown. (b) Bayesian species tree inference using a diffusion model with SNAPPER without an outgroup. Branch lengths represent the number of expected substitutions per segregating site and numbers at nodes are posterior probabilities.

We removed 49 samples identified as genetically admixed in the ADMIXTURE analysis (Table S1) from our phylogenomic analyses (and note that an IQ‐TREE run that does include these samples shows distorted relationships; Figure S3). The concatenated maximum likelihood analysis with IQ‐TREE (Figure 4) and the gene‐tree summary analysis with wASTRAL (Figure 5a) both recover the same topology with high support. The normalised quartet score, which reflects how well gene trees align with the species tree, is 0.54, indicating substantial gene tree discordance. The first lineage to split from the remainder is *veluchiensis*, followed by ‘Vlasina’. Next, there is a bifurcation between *reiseri* and a sister group formed by *apuanus* and *alpestris* (Figures 4 and 5a). The species‐tree estimation with SNAPPER (Figure 5b) differs from that recovered by IQ‐TREE and wASTRAL, suggesting the basal split is between the clade (*apuanus*, *alpestris*) and the clade (*veluchiensis*, (‘Vlasina’, *reiseri*)). However, the effective sample sizes of the SNAPPER analysis are generally low (<< 100).

### 3.6 Molecular Dating

For our time‐calibrated phylogenies, treePL (Figure 4a) consistently recovers older dates and narrower confidence intervals compared to RelTime (Figure 4b). Average divergence times for treePL and RelTime are given before and after the slash. The basal bifurcation in the alpine newt *veluchiensis* and the remainder is dated at c. 19.2/15.1 Ma. Next ‘Vlasina’ splits off at c. 18.3/8.8 Ma, followed by *reiseri* at c. 15.8/6.5 Ma. The crown of the clade comprising *apuanus* and *alpestris* is dated at c. 12.2/5.0 Ma. The crown of *alpestris*, the clade comprising the Central+Western Europe and Spain nuclear DNA subgroups, is dated at c. 9.2/4.1 Ma. The crown of *reiseri* is dated at c. 13.4/6.5 Ma and the Northern Balkan and Southern Romania nuclear DNA subgroups split off at c. 10.4/5.1 Ma. Within the Central Balkan nuclear DNA subgroup, a Western and Eastern group split off at c. 10.2/5.8 Ma.

### 3.7 Historical Introgression

**FIGURE 6:**
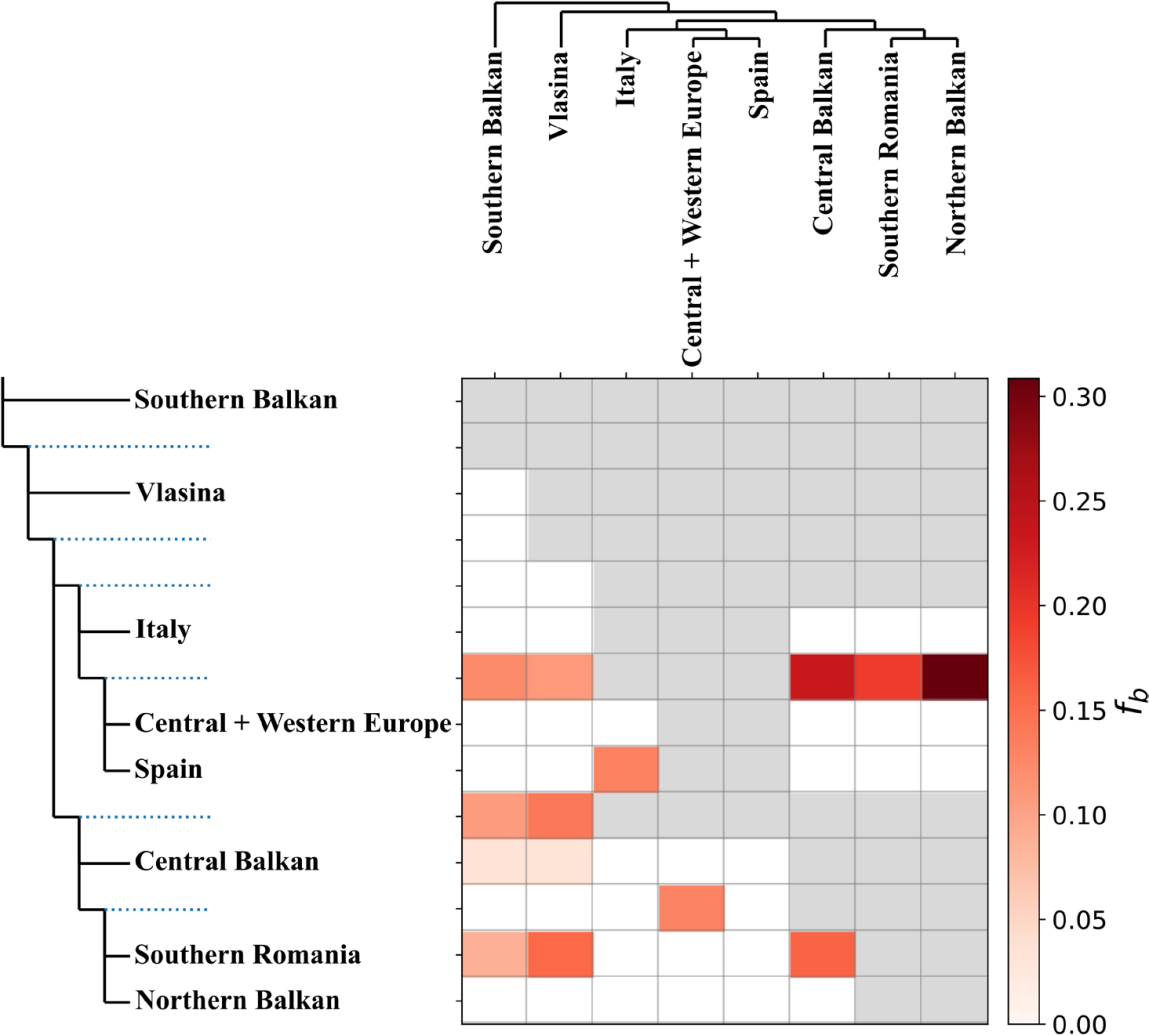
Dsuite f‐branch (fb) heatmap of the alpine newt (*Mesotriton*) cryptic species complex. The colour shading represents the intensity of excess allele‐sharing (i.e., introgression) between tree branches on the *y*‐ and *x*‐axes. No f‐branch statistics could be calculated for the grey cells. The dotted blue lines correspond to the internal branches of the tree.

**FIGURE 7:**
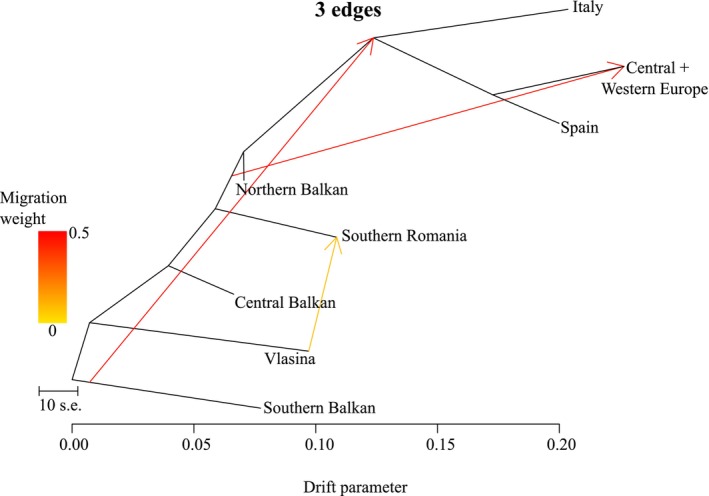
TreeMix admixture graph of the alpine newt (*Mesotriton*) cryptic species complex with three migration edges. Coloured arrows indicate migration weight and directionality of inferred introgression. The scale bar reflects genetic drift from ancestral to extant populations. One out of ten iterations is shown; the other nine can be consulted in Figure S4.

The Dsuite (Figure 6; Table S5) and TreeMix (Figure 7; Figure S4) analyses suggest localised but non‐trivial historical introgression within the alpine newt. In Dsuite, the strongest signal of excess allele sharing is observed between *alpestris* and *reiseri*. Weaker signals are observed between the other lineages. For TreeMix, three migration edges best fit the data (Figure S5). In three out ten replicates, the common ancestor of *apuanus* and *alpestris* is inferred to have inherited a portion of its ancestry from a an ancestral *veluchiensis* population (Figure 7). Additional migration edges are variably placed across replicates (Figure S4), suggesting genetic exchange between nuclear DNA (sub) groups, including the currently allopatric *apuanus*.

### 3.8 Ecological Analyses

**FIGURE 8:**
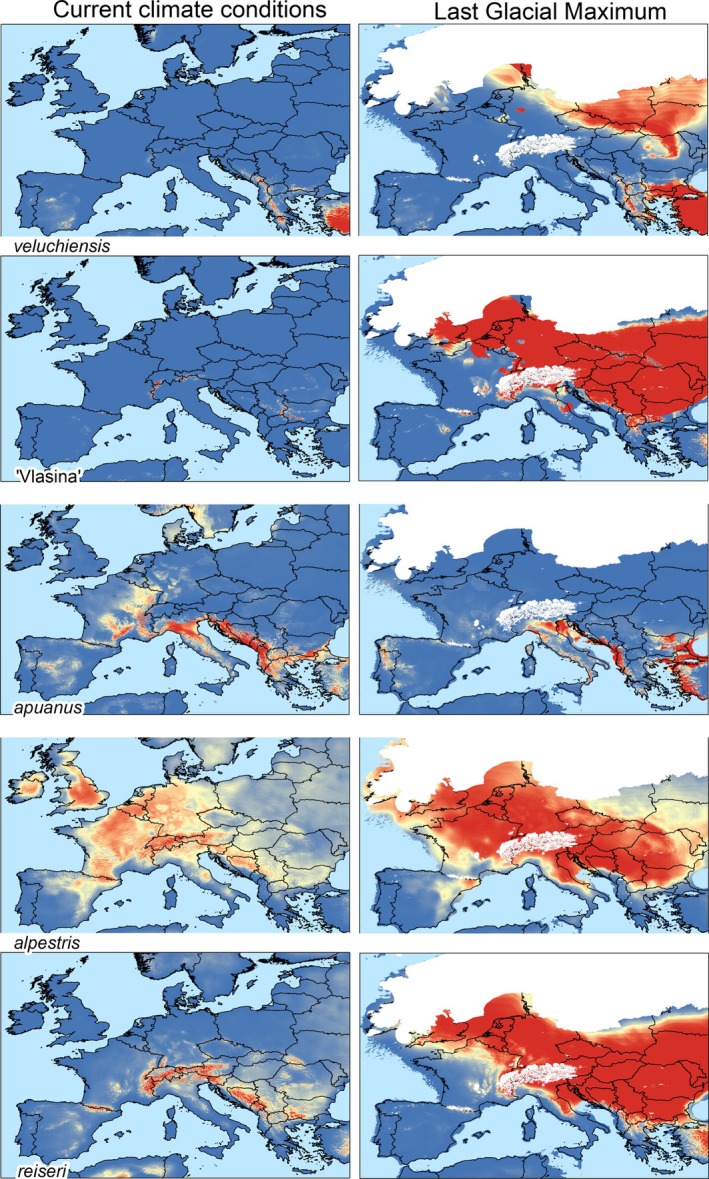
Species distribution modelling of the alpine newt (*Mesotriton*) cryptic species complex. Relative habitat suitability, with warmer colours indicating higher suitability, based on macroclimatic variables for the five nuclear DNA groups, under current climatic conditions and those of the Last Glacial Maximum. The extent of ice sheets at the Last Glacial Maximum (white) follows (Ehlers et al. 2011).

Optimal species distribution models perform significantly better than expected by chance, with mean test AUCs ranging from 0.92 to 0.99. Predicted habitat suitability for one group often extends into the geographic range of another, suggesting shared climatic preferences (Figure 8). During the Last Glacial Maximum, widespread climatic suitability is predicted across northwestern, central and eastern Europe for ‘Vlasina’, *alpestris* and *reiseri*, precluding clear identification of potential glacial refugial areas. Potential glacial refugia for *veluchiensis* and *apuanus* largely coincide with their current distributions.

**FIGURE 9:**
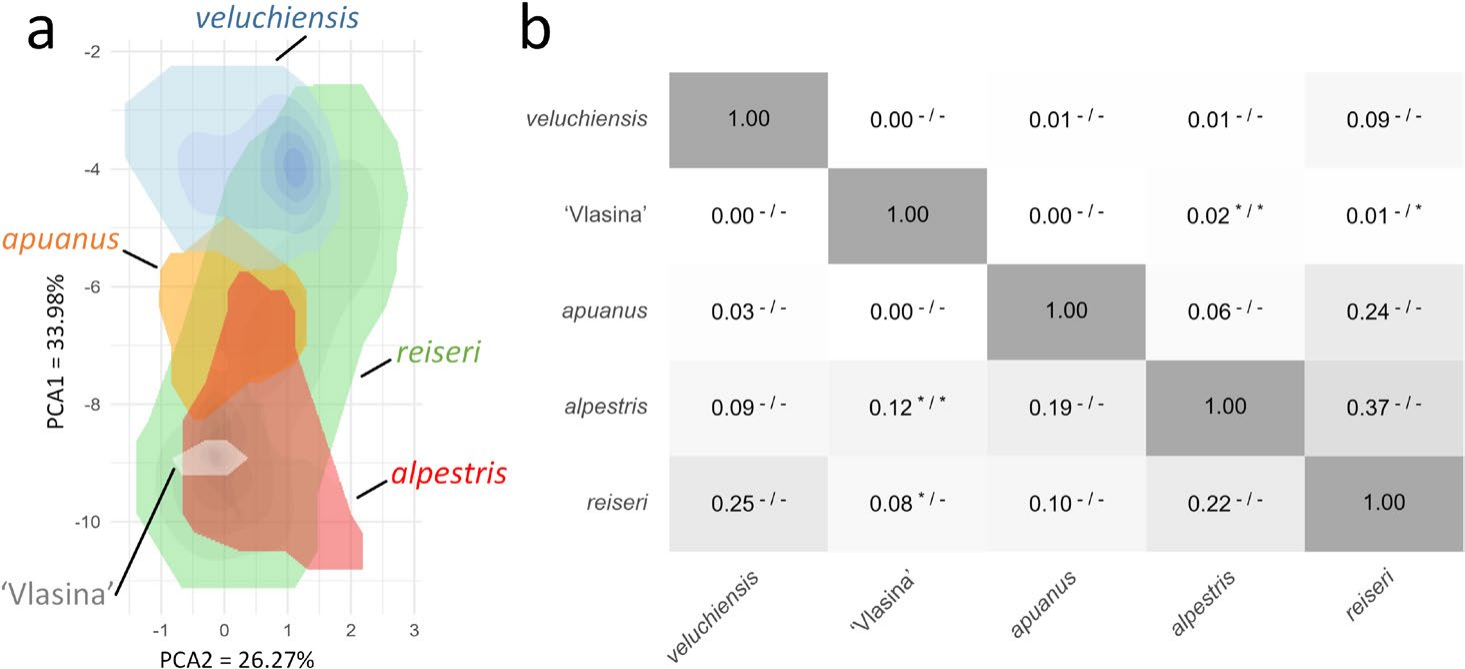
Macroclimatic niches of the alpine newt (*Mesotriton*) cryptic species complex. In (a), the five nuclear DNA groups are shown in two‐dimensional environmental space, defined by the first two axes of a Principal Component Analysis representing climate variation across the study area. Niches are visualised using kernel density estimates, with colour intensity reflecting regions of lower to higher niche occupancy density. In (b), niche overlap is quantified using Schoener's D (above the diagonal) and Hellinger's I (below the diagonal). Values range from 0 (no overlap) to 1 (complete overlap). Niche similarity tests were conducted bidirectionally – comparing each pair in both directions – with significance indicated in superscript next to the overlap scores: An asterisk (*) denotes significant similarity (*p* < 0.05), and a dash (−) non‐significance.

When plotted in environmental space, a considerable overlap between climatic niches becomes apparent. Particularly *reiseri* shows a broad niche that overlaps with all others, while the niches of the remaining groups overlap partially with at least three other groups (Figure 9a). Niche overlap between the different nuclear DNA groups is moderate based on Hellinger's I, ranging between 0 and 0.37 and Schoener's D, ranging from 0 to 0.22 (Figure 9b). However, Schoener's D tends to underestimate overlap when one species displays a smaller or nested niche, as it penalises differences in niche breadth and position, while Hellinger's I emphasises core overlap due to a square root transformation, leading to lower scores if overlap predominates in less‐dense parts of two given niches. Both situations apply to *Mesotriton*. Significant similarity occurs between ‘Vlasina’ and *alpestris* and *reiseri* (and vice versa), as the niche of the former is subsumed in that of the latter two. Similarity tests do not reveal significant dissimilarity between any pair of nuclear DNA groups.

## 4 Discussion

We use target enrichment by sequence capture to disentangle the alpine newt cryptic species complex. Our genome‐wide dataset allows us to delineate distinct genetic clusters, infer the extent of gene flow among them, determine their phylogenetic relationships, and put the extreme mtDNA radiation observed in the genus into perspective. The cold‐adapted alpine newt's range is currently contracted compared to during the Last Glacial Maximum, but from genetic admixture we can infer more extensive geographical contact between genetic clusters in the recent past. On the basis of our new insights, we propose an updated alpine newt taxonomy.

### 4.1 Distinct Species With Contracted Distributions

Guided by our analyses, we recognise five main nuclear DNA groups in the alpine newt, showing geographically limited (at the scale of tens of kilometres) genetic admixture between them, that we consider distinct species: (1) the Southern Balkan *veluchiensis*; (2) a ‘Vlasina’ lineage from the Vlasina Plateau in eastern Serbia and the extreme west of Bulgaria; (3); *apuanus* from Italy; (4) *alpestris* from Central+Western Europe & Spain; and (5) *reiseri* from the Balkan‐Carpathian region (Figure 1a). Some of these species are currently geographically isolated (Figure 1a), even though they show evidence of recent contact through genetic admixture (Figure 3; Table S1). This can be explained by a historically dynamic distribution range, in the past c. 2.5 million years driven by the glacial–interglacial cycles during the Pleistocene Ice Age. Indeed, the alpine newt's bioclimatic niche is conserved (Figure 9) and its current, interglacial, distribution is contracted compared to that of the previous glacial period (Figure 8).

While we observe genetic admixture between ‘Vlasina’ and *reiseri* in the south of Serbia (Figure 3; Table S1), the alpine newt range appears to be highly fragmented here (Vukov et al. 2013). Similarly, in northwestern North Macedonia and the extreme south of Kosovo, we see genetic admixture between *reiseri* and *veluchiensis*, whereas currently the two species appear to be allopatric, with no alpine newt records from most of Kosovo (Tomović et al. 2018). We reveal two relatively narrow hybrid zones between *reiseri* and *alpestris* on the western and eastern sides of the Pannonian Basin (from which alpine newts are currently absent; Figure 1a). Furthermore, we see genetic admixture in several localities far removed from these contact zones (Sillero et al. 2014; Figure 3; Table S1). These instances of genetic admixture, away from current contact zones, presumably reflect an overall interglacial range reduction for the different alpine newt species, in line with our species distribution models (Figure 8) – the shadow of former hybrid zones in a now more fragmented alpine newt range.

### 4.2 ‘Vlasina’ Ghost Lineage?

#### 4.2.1 Yes and No

We confirm that a highly localised and distinct genetic cluster inhabits the Vlasina Plateau in eastern Serbia and the extreme west of Bulgaria (Figure 1a), a particularly biodiverse region, functioning as a glacial refugium and area of endemism (Ranđelović et al. 2010; Tot et al. 2015). A highly diverged alpine newt mtDNA lineage (Figure 1b) has long been known to be endemic here (Sotiropoulos et al. 2007), but was suggested to represent an mtDNA ghost lineage, not reflected in the nuclear genome (Recuero et al. 2014). However, the number of nuclear DNA markers studied (*n* = 2) was arguably too limited to properly disentangle the alpine newt species complex (not only the ‘Vlasina’ lineage). Our phylogenomic analysis reveals that ‘Vlasina’ does in fact represent a distinct alpine newt species, but one that is more deeply nested within the alpine newt than mtDNA would suggest. As a consequence, our molecular dating analysis based on nuclear DNA recovers a more recent date for the crown of the alpine newt than was previously suggested based on mtDNA (c. 8–18 Ma compared to c. 20 Ma; Recuero et al. 2014; Figures 1b and 4). This mito‐nuclear discordance fits a scenario in which mtDNA was captured from an ancient alpine newt lineage, that was mostly replaced at the nuclear DNA level through genetic swamping by another alpine newt lineage (Dufresnes et al. 2020; Hinojosa et al. 2019; Hogner et al. 2012).

### 4.3 Intraspecific Range Fragmentation

We observe discontinuous ranges in several alpine newt species. In *alpestris*, a gap of c. 500 km separates the allopatric Spanish range section from the nearest localities belonging to the Central+Western Europe nuclear DNA subgroup in France. Yet, we observe ‘genetic admixture’ in localities c. 1500 km removed from the Spanish range section, in Italy, Slovenia and Hungary (Figures 1a and 3). This region has been posited as a refugium for *alpestris* (Robbemont et al. 2023; Sotiropoulos et al. 2007). We interpret this as ancestral polymorphism, maintained in a population that, in a first ‘out of the Balkans’ expansion, sourced the now relictual Spanish range segment, and later, in a second ‘out of the Balkans’ expansion, sourced the Central+Western Europe range segment. In western Europe we see relatively minor ‘genetic admixture’, in line with genetic bottlenecking after population expansion from a small source. Species distribution modelling actually supports a wider availability of suitable habitat for *alpestris* at the Last Glacial Maximum compared to now, but with a discontinuous range in the northern Iberian Peninsula (Figure 8).

The range of *reiseri* is severely fragmented (Cogălniceanu et al. 2013; Naumov et al. 2020; Sillero et al. 2014; Vukov et al. 2013), in line with a considerable reduction in suitable habitat in the current interglacial, compared to the previous glacial period (Figure 8). This particularly affects the Central Balkan nuclear DNA subgroup. The Southern Romania nuclear DNA subgroup is separated by over 350 km from its sister lineage, the Northern Balkan nuclear DNA subgroup (Figure 1a). A broad transition zone characterises these Northern and Central Balkan nuclear DNA subgroups, not only where they meet, but also in isolated distribution relicts (Figure 3b).

While our study focuses on the main genetic structure in the alpine newt, we can see some evidence for genetic structure within species and nuclear DNA subgroups. For example, our IQ‐TREE analyses reveal a relatively deep east versus west split within the Central Balkan nuclear DNA subgroup of *reiseri*. This split – albeit with a broad transition zone in between – is supported in the ADMIXTURE analysis under a higher *K* value of 9 (Figure S2). An east versus west divide is observed in mtDNA as well, but is positioned further westwards (the purple ‘western Balkan mtDNA’ clade in Figure 1). This mismatch could be explained by a dynamic distribution, with the western clade (as defined by nuclear DNA) expanding at the expense of the eastern one (Buggs 2007; Currat et al. 2008; Wielstra 2019).

### 4.4 Phylogenetic Discordance and Historical Introgression

The phylogenetic relationships based on nuclear DNA strongly deviate from those suggested by mtDNA (Recuero et al. 2014; Robbemont et al. 2023; Sotiropoulos et al. 2007; Šunje et al. 2021). While mtDNA suggests the presence of an east versus west split in the alpine newt (ignoring the enigmatic Vlasina mtDNA lineage, see Discussion, Section 4.2), nuclear DNA shows that the bulk of genetic diversity is situated in the Balkan Peninsula (Figures 4 and 5a). This pinpoints the Balkan Peninsula as the centre of origin for the alpine newt, which makes sense considering that its closest relatives, the banded (*Ommatotiron*) and mountain (*Neurergus*) newts are distributed in the near east (Kalaentzis et al. 2025; Koster et al. 2025; Rancilhac et al. 2021).

Within *reiseri*, the relationships among nuclear DNA subgroups deviate from those suggested by mtDNA (compare Figure 1b to Figures 4 and 5), and the distinct Northern Balkan and Southern Romania nuclear DNA subgroups do not correspond to reciprocally monophyletic mtDNA clades (Figure 1; Recuero et al. 2014), illustrating the increased phylogenetic resolution obtained with nuclear DNA. In the south of the range of the Northern Balkan nuclear DNA subgroup, we observe mtDNA typical of the Central Balkan nuclear DNA subgroup (the light blue ‘central Balkan mtDNA’ clade in Figure 1). This may suggest that mtDNA of a formerly more widespread Central Balkan nuclear DNA subgroup was ‘left behind’, as it was displaced through hybridisation when the Northern a Balkan nuclear DNA subgroups expanded its range (Buggs 2007; Currat et al. 2008; Wielstra 2019). An additional mtDNA clade in the Central Balkan nuclear DNA subgroup (the purple ‘western Balkan mtDNA’ clade in Figure 1) is poorly reflected in the nuclear DNA (see Discussion, Section 4.3).

For the nuclear DNA‐based phylogeny, the three phylogenetic methods employed do not fully converge on the same topology (Figures 4 and 5). The relatively low normalised quartet score in our ASTRAL analysis reflects substantial gene tree discordance, suggesting that extensive incomplete lineage sorting and/or historical gene flow characterises the evolutionary history of alpine newts (Morales‐Saldaña et al. 2024). This presumably explains (next to the low effective sample sizes) the different topology found with SNAPPER (Figure 5b) compared to IQ‐TREE and wASTRAL (Figures 4 and 5a). In other newt systems (Koster et al. 2025; Mars et al. 2025), SNAPPER was suggested to be relatively susceptible to being compromised by historical gene flow – which we detect in the alpine newt with Dsuite (Figure 6) and TreeMix (Figure 7). In this light, we interpret the IQ‐TREE/wASTRAL topology as more robust.

### 4.5 Taxonomic Recommendations

The alpine newt has a particularly convoluted taxonomic history (Frost 2025). It was formerly treated as part of a broadly defined newt genus *Triturus*, but molecular data proved this treatment untenable, as it would render *Triturus* severely paraphyletic (Caccone et al. 1994; Rancilhac et al. 2021; Titus and Larson 1995). As a consequence, *Triturus* was more narrowly defined and restricted to the crested and marbled newts. Multiple genus names were resurrected to accommodate other newt clades, and the alpine newt was placed in the monotypic genus *Mesotriton* (García‐París et al. 2004). Controversially, the rather grandiose name *Ichthyosaura* was later resurrected to refer to the alpine newt (Schmidtler 2009; Speybroeck et al. 2010), but recently a case has been made to consider this name a synonym of the fire salamander, *Salamandra salamandra* (Mutz and Böhme 2025).

Within the genus *Mesotriton*, taxonomic instability remains in terms of the number of species it comprises. The incomplete picture previously provided by mtDNA (Recuero et al. 2014; Robbemont et al. 2023; Sotiropoulos et al. 2007) has not prevented some authors from proposing to split the alpine newt into multiple species (Raffaëlli 2018). However, the general consensus has been to wait for a phylogenomic study before implementing taxonomic change (Speybroeck et al. 2020). Moreover, there is growing consensus that molecular species delimitation should not only rely on genetic divergence but also – if applicable – on reproductive barriers, which are required to prevent diverging lineages from merging back, noting that both can be evaluated in concert with genomic data and extensive geographic sampling, as done in the present study (Dufresnes et al. 2023; Vences et al. 2024).

In this respect, the five main nuclear DNA groups in the alpine newt make appropriate candidate species under the biological species concept because: (1) in their areas of secondary contact, genetic admixture remains limited, despite the dense sampling, suggesting effective intrinsic (hybrid incompatibilities) and/or extrinsic (ecological differentiation) reproductive barriers, comparable to what is observed between recognised species of Palearctic amphibians, including caudata (Hauswaldt et al. 2011; Kalaentzis et al. 2023; Kazilas et al. 2024; Mattoccia et al. 2011; Wielstra, Burke, Butlin, and Arntzen 2017; Wielstra, Burke, Butlin, Avcı, et al. 2017; Wielstra et al. 2018) and anura (Ambu et al. 2025; Dufresnes et al. 2021; van Riemsdijk et al. 2023). Furthermore, (2) their deep phylogenomic divergence (> 5 Ma) is older than many confirmed species of Palearctic amphibians, showing limited contemporary gene flow at range margins, including in caudata (≥ 5 Ma; Gippner et al. 2024; Koster et al. 2025; Wielstra 2019) and anura (≥ 3–6 Ma; Ambu et al. 2025; Dufresnes et al. 2021). While divergence time is not a direct measure of reproductive isolation, it has been shown to correlate with it (Dufresnes et al. 2021), and can thus be used as an ad hoc criterion, applicable to allopatric lineages. Along the same line, the nuclear DNA subgroups identified are best treated as subspecies.

Therefore, we here provisionally consider the five main nuclear DNA groups in the alpine newt as five distinct species. Applying available taxonomic names under the principle of priority, we propose the following taxonomic revision:

1. *Mesotriton veluchiensis* (Wolterstorff, 1935) – Southern alpine newt.

2. *Mesotriton* sp. – Vlasina alpine newt.

There is no name available for this species; it will be described elsewhere.

3. *Mesotriton apuanus* (Bonaparte, 1839) – Apennine alpine newt.

4. *Mesotriton reiseri* (Werner, 1902) – Reiser's alpine newt.

While this species includes three to four genetically distinct groups, recognising these at the subspecies level is not straightforward. These groups show broad genetic transition zones and type localities of available names are, based on geography, expected to be genetically admixed.

5. Mesotriton alpestris (Laurenti, 1768) – Northern Alpine Newt

The allopatric Spanish population is genetically distinct and we propose it to be treated as subspecies: Iberian alpine newt *M. a. cyreni* (Wolterstorff, 1932). Other proposed subspecies do not represent reciprocally monophyletic groups (Frost 2025; Lužnik et al. 2011; Vörös 2022; Vörös et al. 2021), so we advise against their use.

The identification of hybrid zones between the species in the Balkans opens an exciting venue to study the nature of reproductive barriers in a comparative framework, for example through explicit transect analyses. Moreover, whether phenotypic traits contribute to their speciation and associate to the documented differences in occupied climatic envelopes will be important to assess in future work. Although morphological similarity has kept cryptic species hidden until recently, the growing accessibility of genetic data is rapidly exposing them (Bickford et al. 2007; Espíndola et al. 2016; Fišer et al. 2018; Hending 2025; Struck et al. 2018). Now, with the benefit of hindsight, it might be possible to uncover consistent morphological differences between cryptic species (Hending 2025; Üzüm et al. 2019). We recommend such a study is conducted for the alpine newts. A proper understanding of biodiversity, reflected by an accurate taxonomy, is important for effective conservation (Turvey et al. 2019). Genomic phylogeography provides the tool to obtain the necessary insights.

## Author Contributions

S.K., A.T., W.B. and B.W. designed the research; S.K., A.T., W.B., J.A., W.Ba., D.C., A.C., D.Co., M.C., M.C.V., C.D., J.F., D.J., D.K., S.L., I.M.‐S., B.N., M.P., D.S., B.S., K.S., F.S., D.S., E.M., M.S., E.V., J.V., A.Z., and B.W. performed the research; S.K., A.T., W.B. and B.W. analysed the data; S.K., A.T., W.B. and B.W. wrote the paper, which was improved by J.A., W.Ba., D.C., A.C., D.Co., M.C., M.C.V., C.D., J.F., D.J., D.K., S.L., I.M.‐S., B.N., M.P., D.S., B.S., K.S., F.S., D.S., E.Š., M.S., E.V., J.V., and A.Z.

## Funding

The authors have nothing to report.

## Disclosure

Benefit‐Sharing Statement: The research is relevant to a priority concern (the conservation of the organisms being studied) and the generated data are shared with the broader scientific community on public databases as described above. A research collaboration was developed between scientists from several countries, and all collaborators are included as co‐authors.

## Conflicts of Interest

The authors declare no conflicts of interest.

## Supporting information


**Figures S1–S5:** mec70300‐sup‐0001‐FiguresS1‐S5.pdf.

**Tables S1–S5:** mec70300‐sup‐0002‐TablesS1‐S5.xlsx.

## Data Availability

GenBank Accession numbers for mtDNA haplotypes are in Table S4; Target sequence capture data are deposited in the NCBI GenBank SRA under BioProject PRJNA1304897; All scripts utilised can be found on Zenodo: https://zenodo.org/records/17135911.
