# Supplementary material for: Five Hidden Species in a Widespread European Vertebrate: Disentangling the Alpine Newt Cryptic Species Complex Through Genomic Phylogeography
Source: Mol Ecol. 2026 Mar 9;35(5):e70300. doi: 10.1111/mec.70300 (PMC12970581; doi:10.1111/mec.70300)
Supplement: Supplementary file 1 — Figures S1–S5: mec70300‐sup‐0001‐FiguresS1‐S5.pdf. [file MEC-35-e70300-s001.pdf]

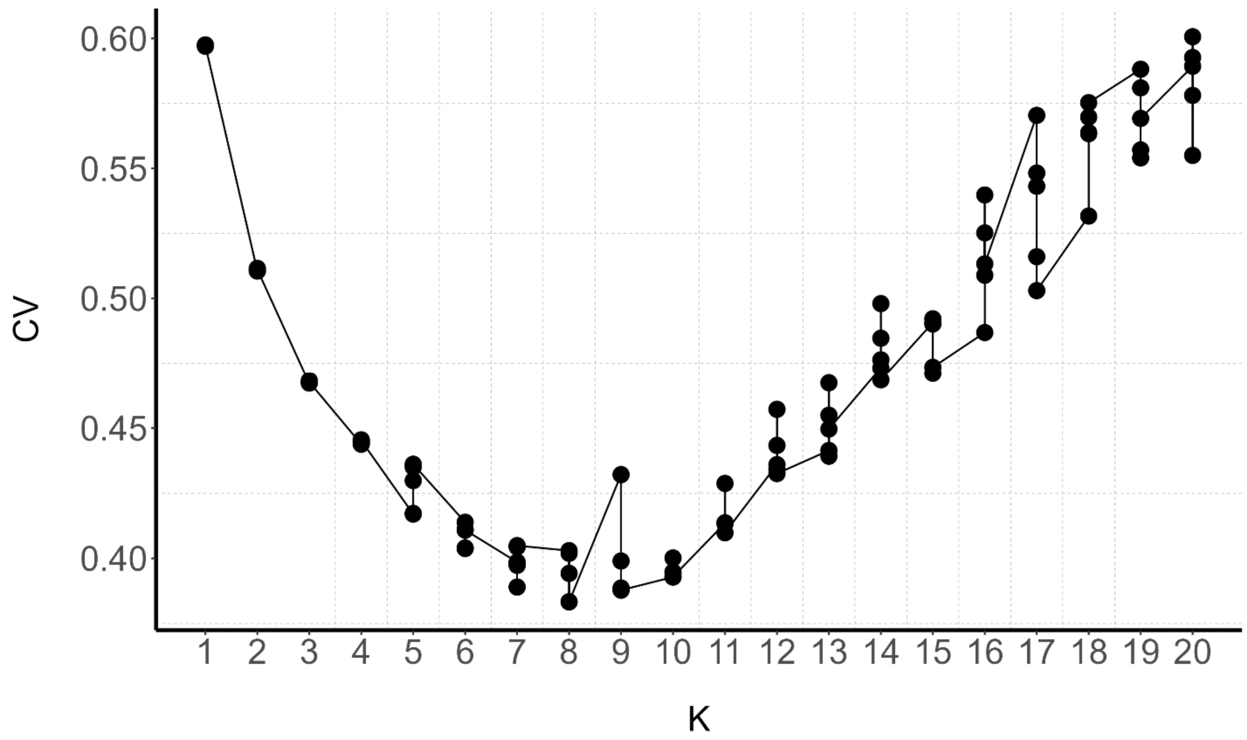

Fig. S1. Cross-validation plot for different numbers of ancestral gene pool (K) of the alpine newt (*Mesotriton*) cryptic species complex.

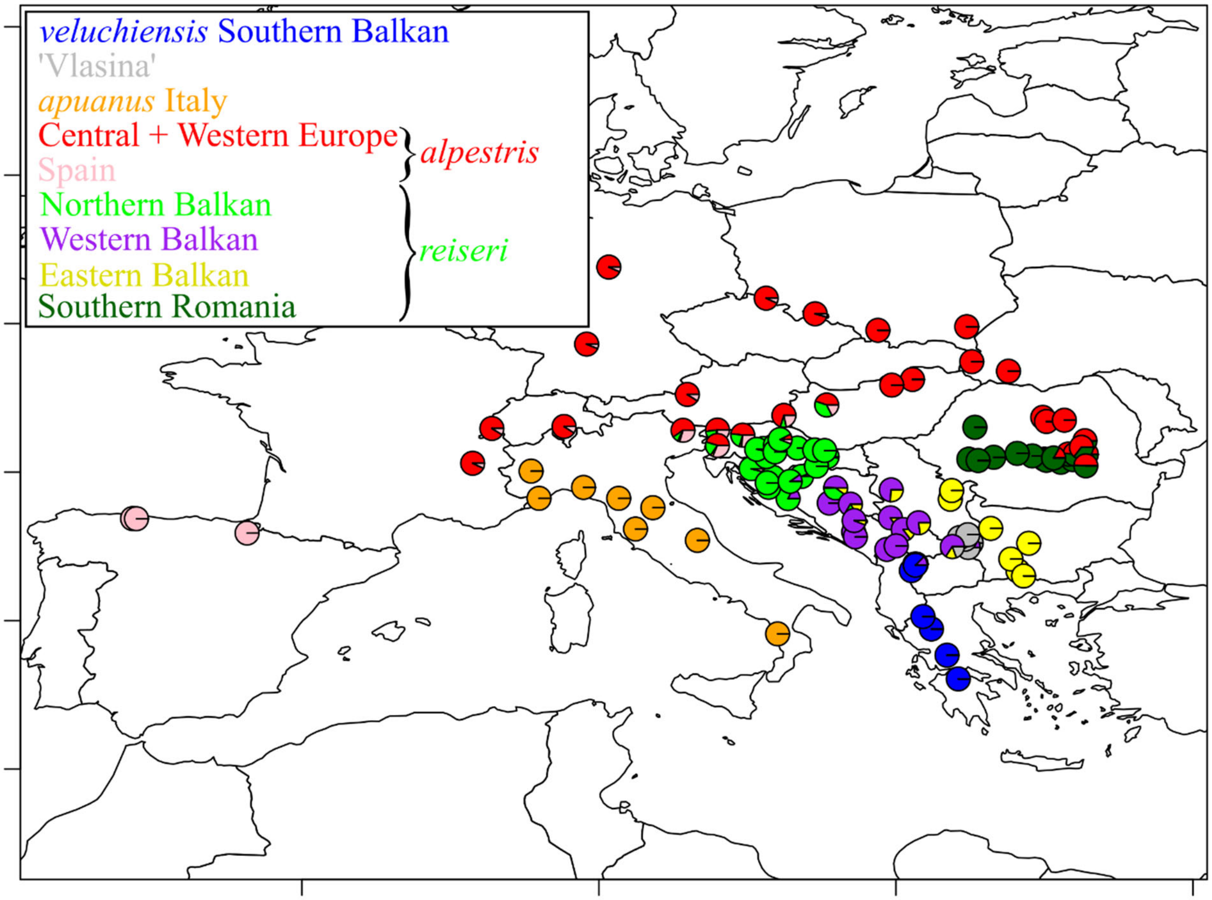

Fig. S2. ADMIXTURE analysis of the alpine newt (*Mesotriton*) cryptic species complex. The number of ancestral gene pools (K) displayed is 9, a value that with a cross-validation error than the preferred K = 8 (Fig. S1), which splits up the Central Balkan nuclear DNA subgroup of reiseri in a Western and Eastern Balkan component. Braces indicate the nuclear DNA subgroups that together correspond to *alpestris* (top) and *reiseri* (bottom). See Table S1 for details.

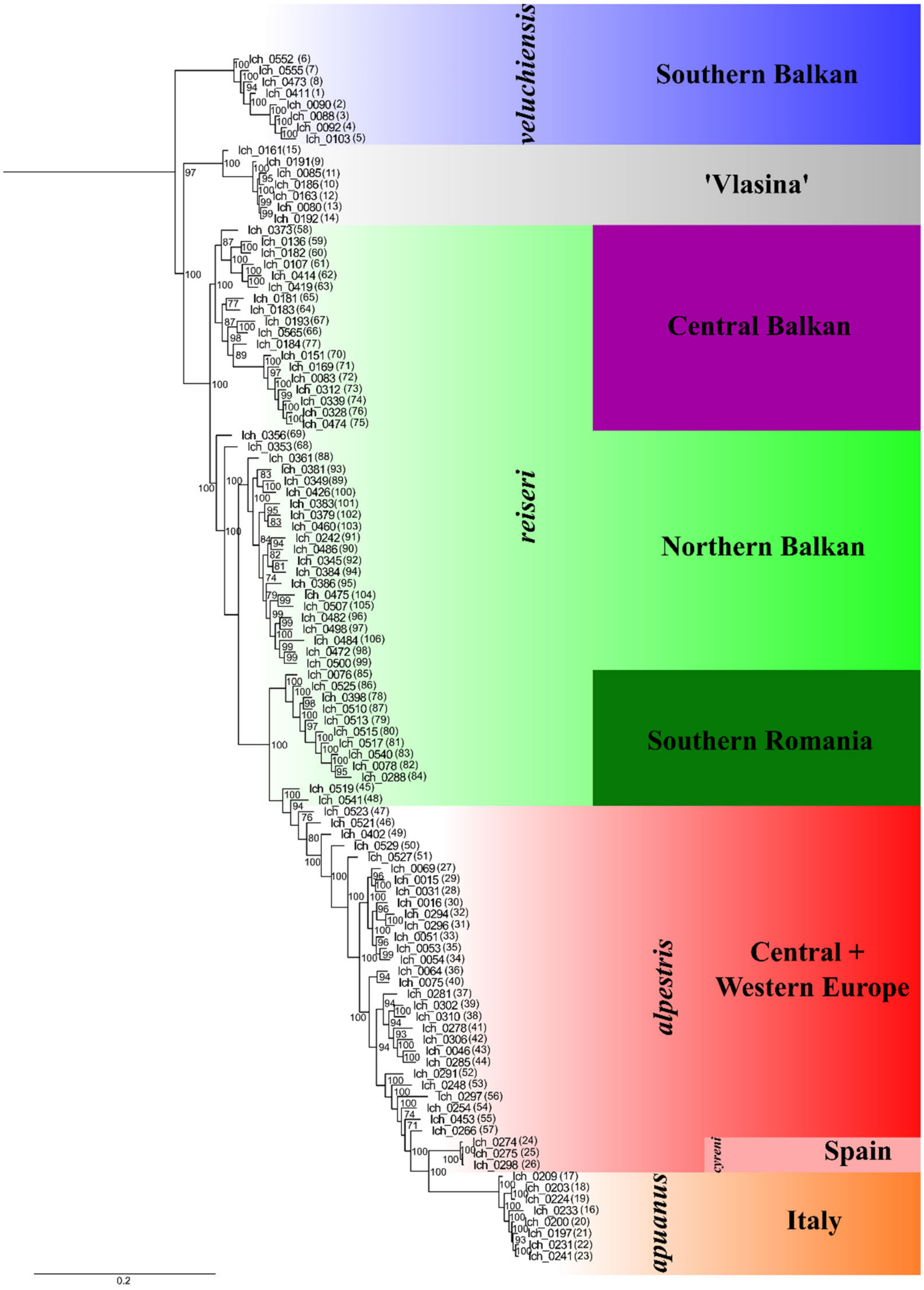

Fig. S3. Phylogeny of the alpine newt (*Mesotriton*) cryptic species complex based on maximum likelihood inference of concatenated data with IQ-TREE, including individuals identified as genetically admixed. This run is based on 71,228 SNPs across 6,892 targets. Values at nodes are bootstrap support values. The scale bar represents the number of expected substitutions per site. Labels correspond to Table S1.

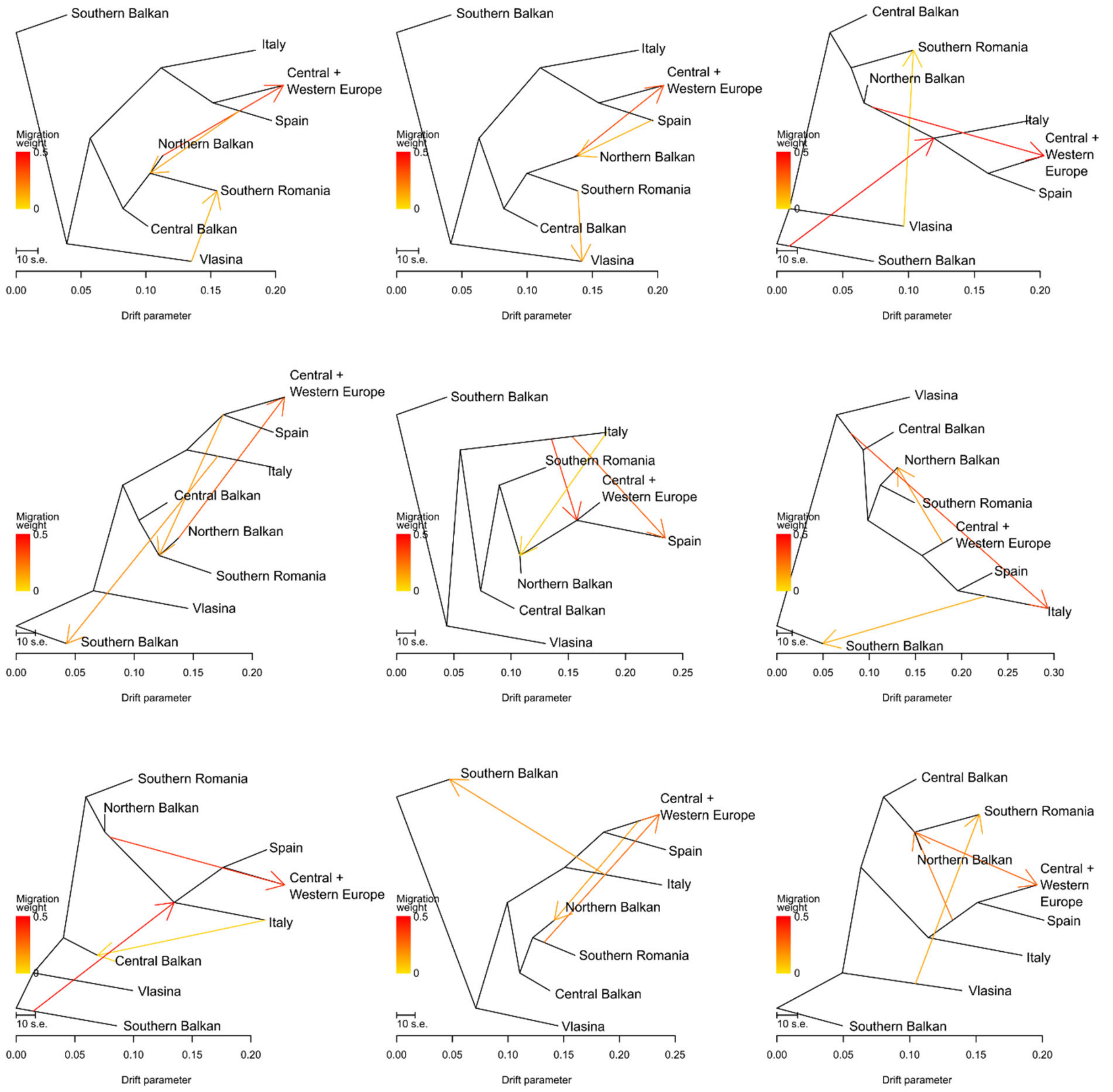

Fig. S4. The other nine iterations of TreeMix admixture graphs of the alpine newt (*Mesotriton*) cryptic species complex with three migration edges. Colored arrows indicate migration weight and directionality of inferred introgression. The scale bar reflects genetic drift from ancestral to extant populations.

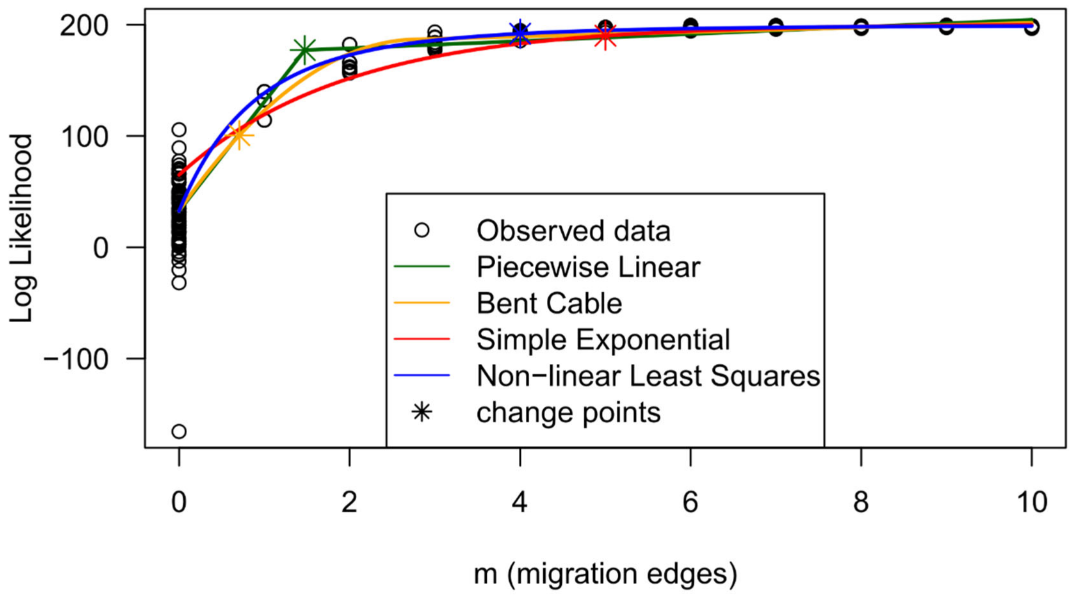

Fig. S5. Selection of the number of migration edges in TreeMix. Explained variance increases with additional migration edges and reaches a plateau for three migration edges.
